# Confidence Thresholds: Towards an Automated Workflow for Unmarked Wildlife Population Modelling With Camera Trap Data

**DOI:** 10.1002/ece3.74227

**Published:** 2026-08-28

**Authors:** Megan Anschau, Simon Denman, Greg Hocking, Grant Hamilton

**Affiliations:** ^1^ Centre for Environment and Society Queensland University of Technology Brisbane Queensland Australia; ^2^ School of Biology and Environmental Science Queensland University of Technology Brisbane Queensland Australia; ^3^ Centre for Data Science, Queensland University of Technology Brisbane Queensland Australia; ^4^ School of Electrical Engineering & Robotics Queensland University of Technology Brisbane Queensland Australia; ^5^ Game Services Tasmania, Department of Natural Resources and Environment Hobart Tasmania Australia

**Keywords:** automated workflow, camera trap, confidence level thresholds, deep learning ensemble, macropod, wildlife population modelling

## Abstract

While ecologists are exploring the capacity of artificial intelligence to automatically identify wildlife in camera trap and other remote sensing data, it has yet to be commonly adopted in workflows that produce density or abundance estimates and thus many image datasets remain under‐analysed. There is potential to enhance image classification with deep learning ensembles which remain uncommon in wildlife studies, and to synergistically connect the output with population modelling in automated workflows. Here we present a repeatable workflow that connects these elements in a case study of macropods (Bennett's wallaby *Notamacropus rufogriseus* and Tasmanian pademelon *Thylogale billardierii*) in Tasmania, Australia. We analysed a pre‐existing camera trap dataset with no contemporaneous field‐collected data. We used readily available computer vision algorithms to train a deep learning ensemble to classify the most common fauna in the dataset, achieving an overall classification accuracy of 94%. Once reviewed, we connected the output to a hierarchical abundance estimation process via a fully scripted workflow designed for repeat application, including the derivation of covariate data from spatial proxies. We then tested the use of classification confidence levels to subset raw (unverified) automated detections, by replacing the verified detection history with a series of automatically generated versions. We found that a detection history derived from a confidence level of 0.75 produced a highly comparable abundance estimate. Our analysis suggested that correctly identifying empty sampling occasions was more important to the estimation process than correctly identifying all occasions with detections. The potential to feed automated detection output that is subset with a classification confidence threshold into a modelling workflow, in place of costly manual verification, represents a high yield opportunity that is worthy of further investigation. In addition, our ensemble approach provides a tool for ecologists who study underrepresented fauna that occur in less frequently surveyed locations and habitats.

## Graphical abstract

Our study explores the application of confidence level thresholds to automated wildlife detections from camera trap data to generate detection histories that could circumvent the need for costly manual review. Our approach employs a study‐specific deep learning ensemble, which is an under‐utilised approach in ecology. Our scripted workflow connects the automated detection output, both manually reviewed and automatically generated, to an established population modelling process. In our case study, we found that a confidence level of 0.75 produced an abundance estimate in close agreement with the estimate from a full manual review.

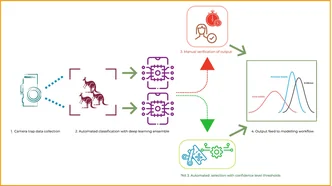

## 1 Introduction

Monitoring is essential to conservation management, particularly in the current biodiversity crisis (Chiatante et al. 2021; Naik et al. 2022). Successful management relies on programs that gather data efficiently and lead to well‐informed and timely interventions (Gentle et al. 2018; Prosekov et al. 2020). Such interventions are frequently based on wildlife population estimates (Linden et al. 2018). The lag that often occurs between data collection and the completion of analyses that deliver population estimates is therefore an impediment to conservation efforts (Whytock et al. 2023). In addition, conservation management requires an understanding of the relationships that exist between wildlife and their habitat (Smith et al. 2022). Monitoring must therefore not only identify population changes but also deliver an understanding of the ecological patterns and dynamics that influence them (Naik et al. 2022; Smith et al. 2022).

Remote sensing technologies are increasingly used for ecological monitoring (Nazir and Kaleem 2021). Remote sensing datasets, however, are often so large that they are difficult for humans to process (Böhner et al. 2023; Lonsinger et al. 2024; Townsend et al. 2021). Ecologists are therefore exploring the capacity of artificial intelligence (AI) to automatically identify objects and patterns that are present in the data (Besson et al. 2022; Nazir and Kaleem 2021). These objects are then classified according to pre‐learned labels, with each classification assigned a confidence score between zero and one to indicate the level of certainty with which the label is applied (Montserrat et al. 2017). Automated object detection is most often performed with deep learning (DL) algorithms (typically either convolutional neural networks (CNNs) or vision transformers (ViTs)), which have a deeply layered architecture that allows them to learn increasingly complex image features from large datasets (Hong et al. 2019). Automated object detection with DL can be enhanced by combining multiple models (trained algorithms) into ensembles that run simultaneously (Dong et al. 2020). While not yet widely adopted in ecology, DL ensembles offer greater predictive and computational power than individual DL models, which can improve the accuracy of automated wildlife detection (Anschau et al. 2022).

One of the most widely‐used remote sensing technologies in ecological monitoring is the camera trap (CT), which enables wildlife to be sampled in a cost‐effective and non‐invasive manner (Delisle et al. 2021; Lu et al. 2022). CT data produces reliable population estimates (Morimando et al. 2016) and a range of models has been developed for this purpose (Gilbert et al. 2020; Palencia et al. 2021). Early CT population models required animals to be individually identified in an approach that mimicked physical capture‐recapture (Palencia et al. 2021). However, fauna characteristics do not always allow for individual identification, so approaches have been formulated for estimating the size of ‘unmarked’ populations. Common examples include a CT distance‐sampling approach (CT‐DS) which incorporates the radius of the CT field‐of‐view (FOV) and CT area of detection, calculated from distance markers placed in the field (Howe et al. 2017). Other approaches are based on calculations of animal activity and speed of movement such as the random‐encounter‐model (REM) (Rowcliffe et al. 2008), random‐encounter‐and‐staying‐time (REST) (Nakashima et al. 2018) and time‐to‐event (Moeller et al. 2018).

While CT surveys are increasingly used to generate wildlife population estimates (Moeller et al. 2018; Palencia et al. 2021), the volume of images captured is a significant impediment to timely analysis (Böhner et al. 2023; Lonsinger et al. 2024). A recent review of CT monitoring in Australia found there was a median lag of 2–3 years between the end of data collection and the publication of analyses (Bruce et al. 2025). There is potential to address this impediment by continuing to enhance the automated classification of wildlife in CT images and connecting the output to population modelling in a streamlined, automated workflow. To date, there is an absence of studies seeking to exploit such potential. This gap was recently highlighted by Zampetti et al. (2024) who explored the potential of using automatically processed CT data for density estimation without human revision, using two publicly available machine learning wildlife detection algorithms, MegaDetector (Beery et al. 2019) via the AddaxAI platform and SpeciesNet (Gadot et al. 2024) via the Wildlife Insights platform. The study presented a semi‐automated workflow in which automated detections were used to generate population density estimates with CT‐DS and REM that achieved promising results, particularly with the CT‐DS approach.

However, CT‐DS and REM both rely on specific information relating to the CT setup to enable FOV angles and distances to be calculated. This information is not always available with pre‐existing datasets which limits their uptake for further analyses, including as the basis for temporal comparison or investigation of non‐target (by‐catch) fauna. There is potential for such datasets to be exploited in hierarchical and mixture models that use repeat sampling to estimate heterogeneous detection probabilities, thereby inferring the underlying (latent) abundance or occupancy, such as those available in the *unmarked* R package (Fiske and Chandler 2011; Royle 2004; Royle and Nichols 2003). Manual verification, however, remains a problem, especially with very large CT datasets.

While unchecked false positive and false negative detections undoubtedly distort the accuracy of automated wildlife counts, it is not known whether the patterns of detection and non‐detection across sampling periods from automated processing mimic the patterns from manual sampling. In this paper, we tested the application of classification confidence thresholds to enable automated wildlife detection output to be connected to an abundance estimation process without manual intervention. We present a case study with a focus on two macropod species (Bennett's wallaby (*Notamacropus rufogriseus*) and Tasmanian pademelon ( *Thylogale billardierii* )) from ~200 K CT images collected in Tasmania, Australia. We first constructed a deep‐learning ensemble to classify a suite of fauna present in the data, including several Australian fauna not commonly studied. To achieve a ‘gold standard’ point of reference, we also manually identified all macropods captured within the study period which allowed us to accurately assess the performance of our ensemble and the downstream products of our workflow. We updated the automated detection record with the results of the manual review, then scripted a workflow to derive the number of independent observations and a detection history suitable for latent abundance modelling in the *unmarked* package. We also used timestamp data from the independent observations to analyse macropod activity. Finally, we generated four subsets of the original automated output (not manually reviewed or updated), retaining only those detections above certain classification confidence thresholds (0.0, 0.5, 0.75, 0.8) and repeated the abundance estimation workflow for each. To assess the performance of the confidence thresholds in our automated workflow, we compared the number of independent observations and latent abundance estimates produced by each subset against the ‘gold standard’ (manual review) outputs, and assessed the relative contribution of true and false sampling results in the detection histories.

## 2 Methods

### 2.1 Background: Macropod Monitoring in Australia

Endemic Australian mammals have been significantly impacted by changes to natural environmental processes since European settlement (Dickman and Pavey 2022). Affected populations include many kangaroo and wallaby species (family Macropodidae, hereafter ‘macropods’). Many small‐bodied macropods have become threatened or gone extinct while many large‐bodied macropods thrive to over‐abundance as a result of human‐induced changes such as permanent water storage, predator removal and vegetation clearing (Read et al. 2021; Wilson and Edwards 2019). Macropod over‐abundance reduces the resources available to sympatric species and disrupts ecosystem functions. Further, peaks of over‐abundance are often followed by devastating crashes that result in widespread mortality as resource availability dwindles (Read et al. 2021; Wilson and Coulson 2021).

Macropod populations in Australia have traditionally been counted in aerial surveys (Lunney et al. 2018) which are constrained by cost, environmental conditions and an inability to detect small‐bodied individuals, and supplementary sampling tends to be negatively biased as kangaroos react evasively to ground‐based observers (Clancy et al. 1997; Moloney et al. 2017). CTs can overcome many of these challenges but their use in macropod studies remains low (Johnson 2019).

### 2.2 Case Study: Macropods in an Agriculture–Conservation Ecotone

Our case study focuses on two species of macropods (Bennett's wallaby and Tasmanian pademelon) on King Island, 80 km off the north‐west coast of Tasmania, Australia. Over‐abundance can be a problem on the island, supported by year‐round availability of pasture, and can cause considerable damage to native vegetation. The Australian Government has recommended further control strategies be implemented (Department of Climate Change, Energy, the Environment and Water 2024). It is therefore timely to consider opportunities to improve the efficacy of monitoring.

We used CT data (216,001 images) collected over 5 months, from October 2022 to March 2023, by the Department of Natural Resources and Environment Tasmania (DNRET) as part of a deer monitoring program. The survey array comprised 36 CTs across an area of ~1000 ha with a mean spacing ± standard deviation (SD) of 566 ± 202 m. This spacing exceeded both target species' home‐range diameter, thereby ensuring sampling independence (Gálvez et al. 2016; Wearn and Glover‐Kapfer 2017). Cameras were placed within 50 m of the centroids of a hexagonal grid, close to sites likely to maximise deer detection such as game trails or browse trees. Cameras were mounted on trees (where available) or star pickets at 0.5–0.6 m to achieve an above ground focal point of 0.6 m at 6 m distance. Distance markers were not used and there were no field‐collected measurements or explicit FOV specifications available. Three CTs did not return data.

**FIGURE 1:**
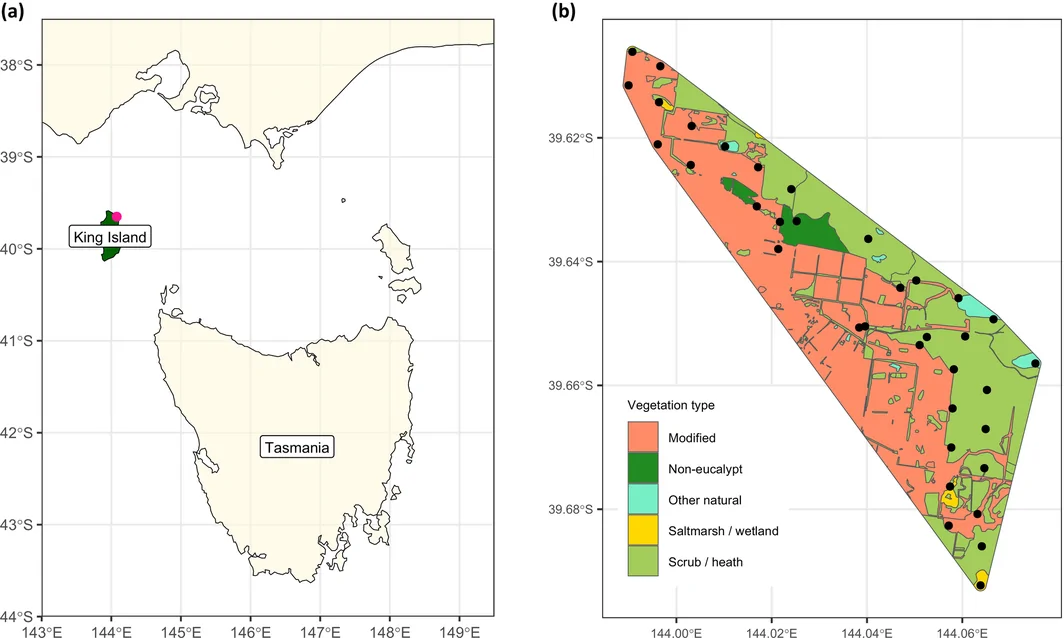
Maps of the CT array deployed from October 2022 to March 2023 showing (a) the proximity of the study area at Lavinia (pink dot) on King Island (dark green polygon) to the state of Tasmania, with mainland Australia to the north, and (b) placement of individual CT sites (black dots) traversing an ecotone between land modified for agriculture and grazing (orange) to the west, and scrub/heath (light green), non‐eucalypt (dark green) and other natural cover (aqua) to the east. Coordinates for two sites in the centre of the array that appear very close have been confirmed as correct.

The survey area traversed an ecotone between agricultural land to the west and Lavinia Nature Reserve on the island's north‐east coast (Figure 1), which is recognised as a wetland of international importance under the Ramsar Convention on Wetlands. The reserve is a regional‐scale biodiversity hotspot which contains tracts of largely unaltered native vegetation that provides significant habitat for a number of threatened wildlife species (Ramsar Sites Information Service, n.d.). We predicted that: (1) macropod activity would be greater in the scrub‐heath and other natural vegetation to the east of the ecotone than in the modified land to the west; and (2) macropod activity in modified land west of the ecotone would be less in daylight hours compared to scrub‐heath and other natural vegetation east of the ecotone where vegetation is likely to provide greater cover.

Images were processed in a high‐performance computing (HPC) environment utilising eight central processing units (CPUs). All other analyses were conducted with R software (R Core Team 2023) in the RStudio environment (Posit Team 2023), including covariate extraction from spatial proxies.

### 2.3 Automated Detection With Deep‐Learning Ensembles

We used transfer learning through a process known as fine‐tuning to train a suite of DL models to recognise all commonly‐occurring fauna in the dataset. Fine‐tuning uses a relatively small corpus of annotated data to train a pre‐trained model on new objects for a new task (Ali et al. 2023; Gui et al. 2024). To achieve this, we iteratively compiled a total of 3854 images from data collected at Lavinia and other locations surveyed across Tasmania as part of the Tasmanian deer management program. The images included 3094 samples across 15 fauna classes (cat, cattle, deer, echidna, hare, macropod, possum, quoll, sheep, Tasmanian devil, wombat, person, turkey, raven and bird‐other) as well as 760 ‘empty’ images containing no fauna. We did not differentiate the two macropod species, which is not uncommon in macropod studies (e.g., Lethbridge et al. 2019; Reid et al. 2020). We manually annotated the images using LabelImg software (Tzutalin 2015), which involved drawing bounding boxes around the fauna in each image and assigning the appropriate labels. As part of the data preparation, a subset of images and annotations was automatically split from the training corpus at a split interval of 1:8 to create an unbiased validation dataset for initial evaluation of model performance.

In accordance with the method described in Anschau et al. (2022), we used the annotated training data to fine‐tune 33 DL models from commonly‐used, ‘off‐the‐shelf’ pre‐trained CNNs (5 each × tiny, medium and large and 3 × extra‐large YOLOv5 v6.0 (https://github.com/ultralytics/yolov5); 5 each × 50‐layer and 101‐layer Faster R‐CNN; 5 × 50‐layer RetinaNet). We initially evaluated the DL models against the validation dataset by calculating the mean average precision (mAP) achieved by each (Figure S1). mAP is equal to the area under a smoothed precision‐recall curve, where precision is the proportion of macropod detections that are correct (Equation 1) and recall is the proportion of present macropods that are correctly identified (Equation 2)

(1) $$\text{Precision}=\frac{\text{True Positives}}{\left(\text{True Positives}+\text{False Positives}\right)}$$

(2) $$\text{Recall}=\frac{\text{True Positives}}{\left(\text{True Positives}+\text{False Negatives}\right)}$$

We then created a suite of 43 DL ensembles by configuring different combinations of the best‐performing models to run simultaneously. We repeated the evaluation process and selected the two best‐performing ensembles for further testing. In brief, the ensemble process runs the component models simultaneously across the data. Detections by each component model are defined by bounding boxes and assigned a confidence score between zero and one. Non‐maximum suppression (NMS) is applied to remove redundant, overlapping boxes where multiple boxes are detected for an object, using the default NMS implementation within the Ultralytics YOLO package. Detections below 0.5 are then discarded and the remaining detections from the individual models are aggregated with overlaps grouped. Where object classes agree, detections with at least 0.8 overlap are progressively merged and a final detection is based on the merged average. A final confidence score is calculated for each detection as the sum of the component scores divided by the number of components in the ensemble (Anschau et al. 2022).

We next ran the two best candidate ensembles across data from several CT sites not seen by the ensemble models during training and validation. Based on initial results, additional images were annotated to address identified limitations with respect to our target species, and fine‐tuning was repeated with the updated dataset. At the end of the fine‐tuning process, we selected an ensemble comprising a large YOLOv5 v6.0 and a medium YOLOv5 v6.0 to process the entire dataset. We evaluated the overall classification accuracy of our selected ensemble according to Equation (3):

(3) $$\begin{array}{c} \text{Accuracy}=\\ \frac{\left(\text{True Positives}+\text{True Negatives}\right)}{\left(\text{True Positives}+\text{True Negatives}+\text{False Positives}+\text{False Negatives}\right)} \end{array}$$

We also processed the dataset with the single best‐performing YOLOv5 and Detectron2 of our trained models to allow us to compare processing time and memory usage.

### 2.4 Detection and Activity Pattern Analysis

Once the dataset was processed, we focussed our analyses on the first 7 weeks of data (17 October to 4 December 2022; 118,663 images) to ensure the closure assumption of our latent abundance models was not violated by the emergence of joeys. We prepared a spreadsheet directly from the automated detection output to record results of the manual review process. It took a team of three people approximately 2 weeks to complete the manual review through a two‐step process. Firstly, each macropod detection was labelled as a true positive, false positive or duplicate. Secondly, macropods incorrectly classified as some other fauna type were labelled as misclassifications, and finally rows were added to the spreadsheet for macropods neither detected nor misclassified (false negatives). Table S1 describes our treatment of the automated detection spreadsheet which would allow our workflow to be repeated in future studies where manual review is undertaken.

We classified detections as independent observations when at least 60 min elapsed between images (Smith 2024). We devised a for‐loop to interrogate sequences with detections less than 60 min apart, to identify instances where triggers were caused by additional individuals entering the FOV. In these cases, the maximum number of individuals occurring in a sequence was added to the count of independent observations. We manually verified these instances to ensure the automated summation worked as intended. From the timestamp data associated with independent observations, we used the *overlap* R package to plot activity in the modified land to the west of the ecotone against activity in the scrub‐heath and other natural vegetation in the east, and calculated a coefficient of overlapping (OVL) to compare the extent to which they agreed. The specific OVL estimator we used (Dhat4) is the most appropriate when activity distributions are based on > 75 observations (Meredith and Ridout 2014). Finally, we calculated relative abundance indices (RAI) for each site by dividing the number of independent observations by the number of operational CT days.

### 2.5 Abundance Estimation Method

We divided the survey period into seven‐day sampling occasions and fit the latent abundance mixture model devised by Royle and Nichols (2003), using the *unmarked* R package (Fiske and Chandler 2011), which incorporates detection/non‐detection data from a single season (*occuRN* function). The model takes in spatially replicated data collected from R sample sites (*i* = 1, 2, …, R) in T repeat sampling occasions (*t* = 1, 2, …, T), where *w*_*it*_ (whether a detection occurred at site *i* in sample *t*) equals 1 for a detection or 0 for non‐detection. The integrated (marginal) likelihood of the data *L(w)* is calculated by incorporating the marginal detection probability masses of the support points for the distribution mixture (i.e., all possible values *k* = 0, 1, 2, …, K) according to Equation (4):

(4) Lw=∏i=1R∑k=0KTwipkwi1−pkT−wifk

where *p*_*k*_ is the detection probability at support point *k*, and *f*_*k*_ *= f(N = k) = Pr(N = k)* where abundance *N* is a random variable. Importantly, the model estimates abundance because the marginal probability masses of the support points represent abundance classes (Royle and Nichols 2003).

### 2.6 Spatial Proxies for Model Covariates

We searched for environmental characteristics likely to influence the presence (abundance process) and detectability (detection process) of macropods. As Bennett's wallaby occurrence is strongly influenced by proximity to streams and ridgelines, and they seek concealment cover in daylight hours (Latham et al. 2023), we selected elevation, slope and distance to water as covariates in the abundance process and included vegetation type in both abundance and detection processes. We sourced relevant spatial proxies (DNRET 2023) and used the *terra* R package to extract covariate values at each CT site (Figure S2).

As rainfall and temperature can significantly influence the detectability component of wildlife abundance estimates (Falaschi 2021), we sourced observational data from the Australian Government Bureau of Meteorology (2026a, 2026b, 2026c) to determine the maximum and minimum temperature, and total rainfall in each seven‐day sampling occasion, which we explored as covariates in the detection process. To the detection process we also added survey effort to account for variation in the number of operational CT days per sampling occasion which can affect the probability of detection (Sollmann 2018), and week number (based on International Organisation for Standardisation rule ISO‐8601). Week number may not always be appropriate in this form, depending on the duration and timing of surveys, but we included it here to test its effect as it may prove useful for temporal comparison of regularly repeated annual surveys. A full list of all covariates and their data sources is provided in Table S2.

### 2.7 Abundance Estimation

We combined the detection history and covariate summaries in an S4 dataframe and standardised all continuous covariates (mean = 0, SD = 1). We built four initial models with the *occuRN* function: all covariates, no covariates (null), observation‐level covariates only (no abundance process), site‐level covariates only (constant detection probability) (Fiske and Chandler 2011). We calculated variance inflation factors (VIFs) which led us to exclude maximum temperature from the detection process (VIF > 4). We then built models to explore combinations of the remaining covariates, including interactions between minimum temperature and rain in the detection process, and elevation and slope in the abundance process. This process resulted in 13 candidate models.

We used the *modSel* function in *unmarked* to compare these models using Akaike Information Criterion (AIC) (Table S3). We selected the model with the lowest AIC as the top model (Equation 5), which also shared the highest coefficient of determination (*R* ^2^ = 0.86) and greatest parsimony. A likelihood ratio test confirmed the model was significantly better at predicting latent macropod abundance than the null (Chi‐squared = 64.68, df = 7, *p* = 1.74e‐11).

(5) $$\begin{array}{l} \text{abundance}\;\left(N\right)\;\sim \text{distance to water}+\text{elevation}+\text{slope}+\text{vegetation type}\\ \text{detection}\;\left(p\right)\;\sim \text{minimum temperature}+\text{effort}+\text{vegetation type} \end{array}$$

We ran the top model with the observed data to generate maximum likelihood estimates (MLE) of the model parameters, then calculated empirical Bayes estimates to predict the conditional distribution of the latent abundance at each site (Royle and Dorazio 2008). We evaluated goodness‐of‐fit by comparing test statistics from the fitted model (sum of square errors (SSE), Pearson's chi‐squared and Freeman Tukey) with test statistics generated from 1000 parametric bootstrap samples (*parboot* function, nsim = 1000) per MacKenzie and Bailey (2004).

### 2.8 Classification Confidence Thresholds for Automated Detection Histories

We tested the use of classification confidence level thresholds to subset the raw (unverified) automated detection output, to automatically generate the binary detection histories required for our abundance estimation workflow. Success with such an approach could circumvent the need for manual review of detections. We tested thresholds of 0.0, 0.5, 0.75 and 0.8. We specifically included 0.75 as it is the default setting used by Timelapse Image Recognition software (Greenberg Consulting Inc. 2026) in relation to MegaDetectorv5, as applied in Zampetti et al. (2024). We then fed the auto‐generated detection histories (collectively DH‐autos, individually DH@0.0, DH@0.5, DH@0.75 and DH@0.8) into our abundance estimation workflow in place of the detection history derived from manually reviewed observations (DH‐MR). We predicted that even without correction for false positives and false negatives, when observations were distilled to detections and non‐detections per sampling unit there would be a confidence level at which there would be sufficient similarity in a DH‐auto to produce a comparable abundance estimate to DH‐MR. To assess this, we first compared the number of independent observations and latent abundance estimates produced by DH‐autos with the estimates from DH‐MR. We then explored the possibility that differences in the distribution of the binary detection [1] and non‐detection [0] labels in DH‐autos influenced the latent abundance estimates. We summarised the number of sites in each sampling week from 194 samples that returned data, where the labels in DH‐autos matched the labels in DH‐MR (match = ‘true’, non‐match = ‘false’). We used chi‐squared tests of independence to determine whether the numbers of true matches in each weekly sample were significantly associated for each pair of thresholds. As the number of matches were strongly associated (no significant difference to potentially influence differences in abundance estimates), we further interrogated DH‐autos, classifying each match as a true positive (matching a DH‐MR [1]) or true negative (matching a DH‐MR [0]) and each non‐match as a false positive (where DH‐MR = [1]) or false negative (where DH‐MR = [0]). We summarised the results for each threshold and tested for significant association with a chi‐squared test of independence.

**FIGURE 2:**
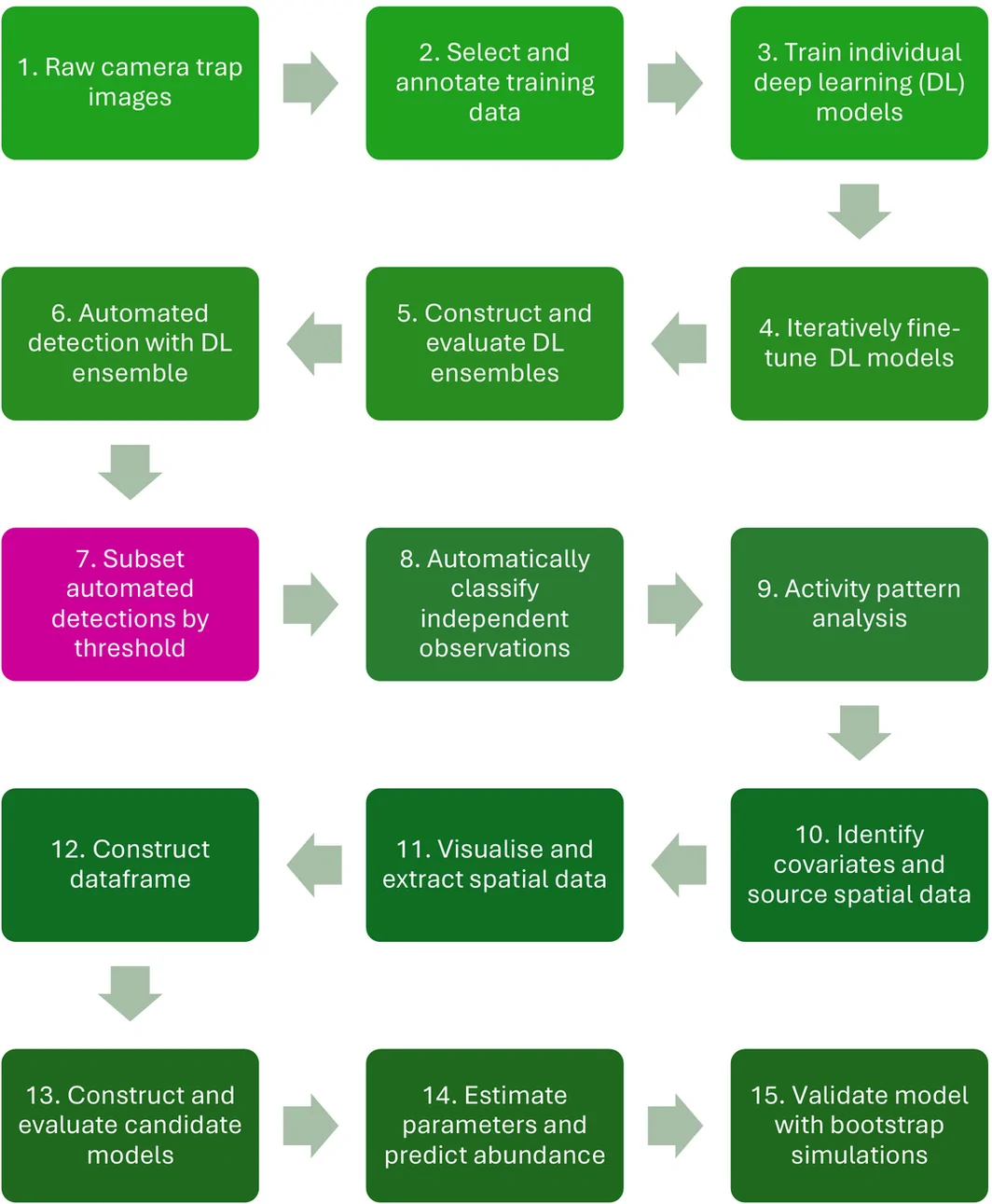
Flow chart showing the methodological steps in our automated latent abundance estimation workflow. Step 7 (pink box) is the point in the workflow where we substituted automatically generated detection histories, derived through the application of classification confidence thresholds, in place of a detection history derived from manually reviewed observations.

The complete workflow is summarised in Figure 2.

## 3 Results

### 3.1 Automated Detection Output and Analysis

A comparison of the time it took our ensemble to process the 216,001 image dataset with the time taken by the single best‐performing YOLOv5 and Detectron2 of our trained models is provided in Table 1. All processes were run in the same multi‐core HPC environment on a distributed Linux cluster utilising eight CPUs and one GPU. The ensemble, which comprised one large YOLOv5 and one medium YOLOv5, ran 1 h and 50 min more (+27%) than the single medium YOLOv5, and both used 1.3 gigabytes (GB) of memory. The single Detectron2 type model, a Faster‐Regional Convolutional Neural Network (F‐RCNN with 101 layers), was more computationally demanding than the ensemble, requiring an additional 1 h and 34 min (+18%) as well as an additional 0.24 GB (+18%) of memory.

**TABLE 1:** A comparison of processing time and memory usage of our selected ensemble compared with the single best‐performing YOLOv5 and Detectron2 type models.

Model/ensemble	CPU (processing) time	Memory usage (GB)
Ensemble: large YOLOv5 + medium YOLOv5	8 h 42 min	1.30
Single: medium YOLOv5	6 h 52 min	1.28
Single: F‐RCNN 101 layers	10 h 16 min	1.54

Automated classification performance metrics were calculated from the results of the manual review. Our DL ensemble achieved an overall classification accuracy of 94%. Precision of at least 90% was achieved at 16 sites, with an average of 82% across 29 sites where macropods were present and 72% including zero‐detection sites. Recall was lower, averaging 54% across all sites, with five sites above 74% and five sites below 40%. A complete summary of the performance metrics is provided in Table S4.

The detection process yielded 2477 independent macropod observations from a total of 1286 CT days. Eleven CTs stopped functioning before the survey period ended. This introduced variation in survey effort which we accounted for in our model's detection process. Four sites with zero detections were all located on the north‐eastern edge of the sampling area, either in or adjacent to modified habitat. More than 100 independent macropod observations were captured at eight sites, with at least 288 at three sites. Mean RAI was 1.8 ± 2.0 (SD) with SE of 0.9. The highest RAI (8.1) occurred in the far south where the sampling area opens onto a large tract of undisturbed scrub‐heath (Table S5).

**FIGURE 3:**
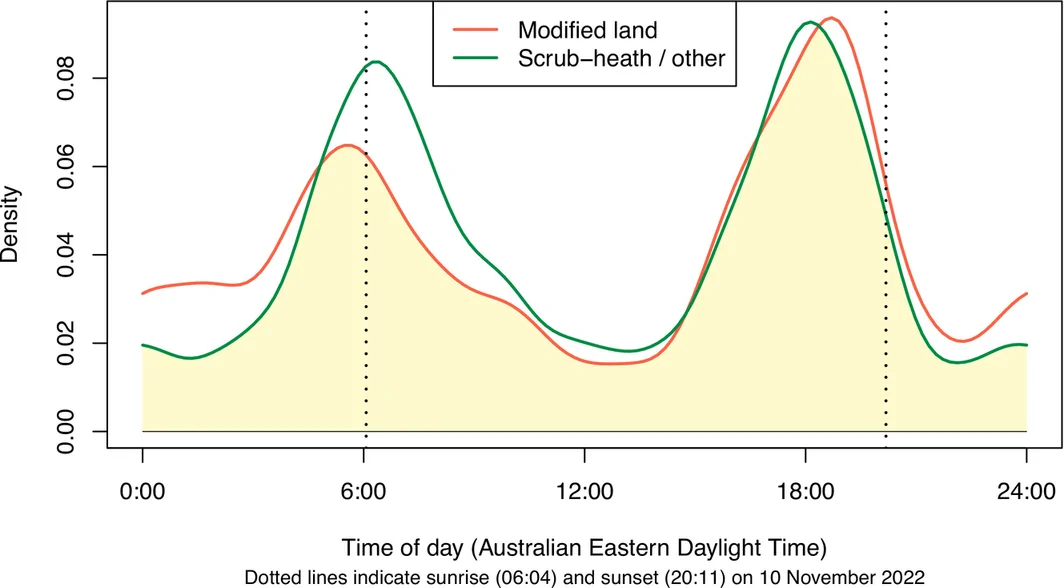
Overlapping activity plots based on independent macropod observations either side of the ecotone at Lavinia, King Island, from October to December 2022, with dotted lines indicating sunrise at 06:04 and sunset at 20:11 on 10 November 2022 (Australian Government Geoscience Australia, n.d.).

Macropod activity across the 7‐week study period was bimodal and strongly crepuscular (Figure 3). There were 630 independent observations in modified land west of the ecotone and 1070 in scrub‐heath/other natural areas to the east, which agreed with our prediction that activity would be greater in unmodified land. The OVL determined there was close agreement (89%) between the two activity distributions. However the overlapping activity plot showed that a greater proportion of the activity in modified land occurred overnight, particularly during pre‐daylight hours, and reached an earlier and much lower pre‐dawn peak, whereas activity in scrub‐heath/other natural vegetation increased to a much higher peak closer to sunrise and remained elevated in early daylight hours, thus confirming our second prediction. Interestingly, the activity peak before sunset was very similar in both environments.

### 3.2 Latent Abundance Estimation

The MLE parameter estimates are shown in Table 2. Slope was the only significant covariate in the abundance process (*p* = 0.037) with increasing slope having a positive effect (+0.347) on macropod abundance. Increasing elevation also had a positive, though non‐significant, effect (+0.117) but abundance decreased with increasing distance from water (−0.104). Vegetation type had the largest effect size, with lower abundance predicted in scrub‐heath/other natural vegetation compared to modified land (−0.406). Further, approximately 52% of detections occurred in scrub‐heath/other natural vegetation (22 sites) with 48% in modified land (11 sites) (Figure S3), which was a highly significant difference in proportions (chi‐squared = 19.121, df = 1, *p* = 1.23e‐05). All covariates in the detection process were highly significant, with detection probability increasing with additional survey effort (+1.544) and decreasing with increases in minimum temperature (+0.18). Detection probability was also higher in scrub‐heath/other natural vegetation compared to modified land (+1.310). The model predicted a total of 142 macropods (SE = 2.09) across all 33 sites, with an average detection probability of 0.48. Average site abundance was predicted to be 4.93 (SE = 2.46) with an average probability of detection of 0.21 (SE = 0.11).

**TABLE 2:** Maximum likelihood parameter estimates and standard errors (base vegetation category = modified land).

	MLE	SE	Z‐score	*p*(>|*Z*|)
Abundance parameters (logarithmic scale)				
Intercept	1.596	0.498	3.207	0.001
Distance to nearest water (m)	−0.104	0.142	−0.732	0.464
Elevation (m)	0.117	0.188	0.621	0.534
Slope (^0^)	0.347	0.166	2.083	0.037
Vegetation: scrub‐heath/other natural	−0.406	0.585	−0.694	0.488
Detection parameters (logit scale)				
Intercept	−1.308	0.641	−2.040	4.13e‐02
Minimum temperature	−0.156	0.182	−0.856	3.92e‐01
Effort	1.544	0.244	6.321	2.60e‐10
Vegetation: scrub‐heath/other natural	1.310	0.803	1.630	1.03e‐01

The empirical Bayes estimates predicted an average latent abundance of 4.3 macropods per site (95% CI = 1.9, 7.7) with a total of 142 macropods (95% CI = 63, 254). Bootstrap simulations showed no evidence of poor model fit (Figure S4).

### 3.3 Classification Confidence Level Threshold Results

We compared the number of independent observations and latent abundance estimates produced by DH‐autos with the estimates from our ‘gold standard’ DH‐MR (Table 3). Comparatively speaking, DH@0.75 performed best, generating estimates that almost matched the DH‐MR estimates (148 vs. 142). The 95% confidence and credible intervals (CIs) were also very similar. DH@0.8 produced an 18% underestimation (117 vs. 142) whereas DH@0.5 and DH@0.0 produced 27% and 70% overestimations respectively (181 and 241 vs. 142). Unsurprisingly, the number of independent observations decreased as the confidence threshold increased. However, none of the counts approached the verified count, reflecting the relatively high number of false negatives in the automated detection output which was heavily influenced by clustering at three sites where there were also high numbers of true positives. In addition, the relative similarities with DH‐MR counts were not reflected in the similarity with DH‐MR abundance estimates. Average detection probabilities varied neither proportionally nor directionally with the DH‐MR values.

**TABLE 3:** Comparison of latent abundance modelling outputs from four detection histories automatically generated with classification confidence thresholds versus outputs from a detection history derived from manually reviewed observations.

Classification confidence threshold	Count of independent observations	Average detection probability	Fitted model latent abundance estimate	95% confidence interval	Estimate from empirical Bayes posterior distribution	Empirical Bayes 95% credible interval
Manual review	2477	0.48	142	(56, 377)	142	(63, 254)
0.0	1964	0.43	241	(121, 492)	241	(115, 394)
0.5	1170	0.33	181	(87, 385)	181	(87, 307)
0.75	1012	0.38	148	(71, 314)	148	(68, 256)
0.8	917	0.39	117	(57, 243)	117	(54, 211)

*Note:* Green cells highlight the close agreement between estimates from DH‐MR and DH@0.75.

A summary of the number of sites in each sampling week (from 194 samples that returned data) where the detection [1] and non‐detection [0] labels in DH‐autos matched the labels in DH‐MR is provided in Table 4. Even though there were considerable differences in the number of independent observations derived by each threshold, there was little difference in the number of [0, 1] matches across sampling units (Figure S5), with all DH‐autos matching DH‐MR labels in at least 85% of the samples. Further, DH@0.0, which produced the least comparable abundance estimate, had the highest number of matches with DH‐MR, while DH@0.75 (the most comparable) was third and DH@0.8 (second most comparable) was last. Chi‐squared tests of independence confirmed the results between each pair of thresholds were highly associated (Table S6), from which we inferred there was no underlying pattern in the spread of detections vs. non‐detections that could influence the latent abundance estimates.

**TABLE 4:** The number of [0] and [1] label matches per weekly sampling unit between each DH‐auto and DH‐MR. The header row shows the number of sites in that week with cameras in operation (sites that returned data).

Sampling unit (*n* sites with data)	Week 1 (33)	Week 2 (31)	Week 3 (28)	Week 4 (28)	Week 5 (27)	Week 6 (24)	Week 7 (23)	Total (194)
*n* [0,1] matches @0.8	30	30	25	24	23	18	14	164 (85%)
*n* [0,1] matches @0.75	30	30	27	23	23	18	15	166 (86%)
*n* [0,1] matches @0.5	30	29	26	23	22	21	17	168 (87%)
*n* [0,1] matches @0.0	30	27	26	26	24	22	19	174 (90%)

Table 5 summarises our interrogation of the matches and non‐matches in conjunction with DH‐MR [0,1] labels. A chi‐squared test of independence showed the association between the values derived from each threshold was significant (*χ* ^2^ = 32.891, df = 9, *p*‐value = 1.395e‐04). While all DH‐autos returned a relatively high number of true positives, this metric was lower for DH@0.75 and DH@0.80. DH@0.75, which came close to matching DH‐MR abundance estimates, and the next‐best DH@0.8, had more false negatives but considerably less (and very few) false positives, particularly compared to DH@0.0 that produced very poor comparative abundance estimates. Conversion of the values to precision and recall metrics highlighted the higher precision coupled with lower recall achieved by DH@0.75 (97%, 85%) and DH@0.8 (98%, 83%). These metrics support an inference that correct identification of empty samples (true negatives maximised and false positives minimised) contributed to more comparable latent abundance estimates.

**TABLE 5:** Comparison of matches and non‐matches between DH‐autos and DH‐MR in conjunction with the verified weekly sampling labels [0,1] such that a match with a [1] = a true positive, a match with a [0] = a true negative, an unmatched [1] = a false negative and an unmatched [0] = a false positive.

Threshold	False negative	True negative	False positive	True positive	Precision	Recall
0.0	5	17	15	157	91%	97%
0.5	18	24	8	144	95%	89%
0.75	24	28	4	138	97%	85%
0.8	27	29	3	135	98%	83%

## 4 Discussion

Our study contributes to the ongoing exploration of advances in computer science for the benefit of ecological study and offers another step towards a fully automated workflow. Despite its capacity to process large volumes of CT data, AI has yet to be commonly adopted in workflows that produce density or abundance estimations (Zampetti et al. 2024). As such, the large volume of images generated in CT surveys remains a significant barrier to timely analysis (Böhner et al. 2023; Lonsinger et al. 2024) and to the broader application of CT data to wildlife population studies (Li et al. 2025; Zampetti et al. 2024). Our DL ensemble approach, which achieved high classification accuracy with a relatively small amount of annotation effort, could particularly benefit ecologists who study underrepresented wildlife in underrepresented environments. We demonstrated this potential through our case study, in which we derived an abundance estimate for a previously unstudied macropod community on an Australian island where an ecologically sensitive RAMSAR‐listed environment meets economically important grazing and agricultural land. A lack of suitable road access meant this area had not previously been surveyed in an annual monitoring program undertaken manually by vehicular spotlight survey.

We note that estimates produced by this type of modelling cannot be interpreted as absolute abundance as the effective sample area covered by the camera trap array can only be approximately described (Royle and Dorazio 2008). Such estimates more accurately represent the unknown (latent) number of individuals available for detection within the collective camera viewsheds (Bollen et al. 2023). The inability to accurately calculate an effective sample area is an unresolved problem for ecologists who use camera trap observations in abundance (and density) models (Bollen et al. 2023; Gilbert et al. 2020). Various methods are employed to address this, such as defining the sample area by drawing a convex polygon around all cameras within the array (e.g., Alexiou et al. 2025) or by summing and potentially extrapolating the estimates from each camera (e.g., Baldwin et al. 2023) or from grid cells superimposed across the array (e.g., Koetke et al. 2024). However, while we acknowledge there is uncertainty as to the effective sample area, the latent abundance estimate derived from our workflow is nonetheless useful as a baseline against which future estimates can be compared. As ecologists we need to remain pragmatic about what can be achieved while continuing to work towards improvement and future advances.

Our use of classification confidence levels to subset raw (unverified) automated detection output represents a potential enhancement to current practice. Even without the inclusion of an automatically generated detection history, the easy repeatability of a fully scripted workflow offers important time savings between CT data collection and the completion of analyses. With automated detection output reported in a fixed‐format summary spreadsheet, a pre‐scripted approach to data wrangling can efficiently transform the large volume of unfiltered detection output (in our case ~145,000 lines comprising 5 months of detections across 15 fauna types) into an input that is suitably formatted for the modelling process. Further, some or all of the environmental covariates extracted in our workflow (elevation, slope, minimum/maximum temperature, rainfall and distance to water) will be relevant to many other vertebrate studies, enhancing the repeatability.

DL ensemble learning remains uncommon in ecological studies (Li et al. 2025). In our study we chose to verify the performance of our DL‐ensemble by manual inspection, which afforded complete confidence in the performance metrics and a sound basis for evaluation of the automatically generated detection histories within the workflow. As the Royle and Nichols (2003) latent abundance model requires only detection/non‐detection per sampling occasion, it would be sufficient to review each occasion only until a single detection was manually verified. In this way, only those sampling occasions in which no target fauna are captured would be fully reviewed. Such an approach would facilitate more rapid estimation from large CT datasets that has utility for adaptive management and temporal comparison. Depending on data ownership and permissions, it may also be possible to reduce the manual review effort by pre‐processing the images through a tool such as MegaDetector (Beery et al. 2019) to identify and remove images containing no fauna prior to the automated detection process (e.g., as in Zampetti et al. 2024).

While more investigation is needed to verify the potential, a confidence threshold of 0.75 in our case study delivered latent abundance estimates and 95% CIs from automated detection output (with performance metrics of 72% precision and 54% recall) that were highly consistent with estimates derived from a full manual verification of all detections across the 7‐week study period. If further study can demonstrate similar results, classification confidence thresholds could provide a means of connecting automated image processing with population estimation processes, bringing ecologists a step closer to a fully automated workflow. We note a recent study by Zampetti et al. (2026) prescribed 0.75 as a confidence threshold for a taxonomic aggregation strategy (proposed by Gadot et al. 2024) which improved the predictive performance of a CNN trained for animal classification.

There are now a number of publicly available deep learning models that have been trained to classify many species of wildlife that will greatly benefit the global community of ecologists who work with camera trap data. However, wildlife classification models are only applicable if they contain the target species and have been trained on data that represents the target environment. The uniqueness of Australia's native fauna is therefore an impediment to successful processing with tools trained for northern hemisphere fauna, such as SpeciesNet, and many Australian species are underrepresented in current CT databases (Bruce et al. 2025). This is a recognised gap as evidenced by initiatives such as The Wildlife Observatory of Australia (WildObs) which is working to establish a continent‐wide database of Australian wildlife (https://www.tern.org.au/wildobs/) and has trained its own version of SpeciesNet so that Australia's unique species can be recognised and accurately classified (Google Research 2026). Another emerging example is the Australian Wildlife Classifier (AWC135) released by the Australia Wildlife Conservancy in February 2026 and trained to recognise 135 species from the Australian mainland (Australian Wildlife Conservancy 2026). However, none of the data used by AWC135 at this stage have been collected from Tasmania which represents a domain gap for our study. Our study is unique in that we utilised a DL ensemble, for which we fine‐tuned a selection of widely‐used CNNs to identify 15 different classes of fauna from a large dataset collected in a previously unsurveyed part of Australia. While we focussed our case study on macropods, our ensemble could have been used for any of the 15 fauna classes (singly or in combination) that it had learned.

Given the above, it remains that publicly available models still require fine‐tuning to new target domains, which is not appreciably different to what we have undertaken in this study by fine‐tuning commonly used, ‘off‐the‐shelf’ computer vision algorithms. In terms of generalisability, one of the strengths of our approach is that it creates a classifier that is optimised for the specific target domains of the study, where such classifiers are not otherwise available. It is recognised that the performance of DL detection models can be improved by training with geographically‐specific data as opposed to data that is broader in coverage (Zampetti et al. 2024). Our method therefore provides an approach for ecologists anywhere in the world who study underrepresented fauna that occur in less frequently surveyed locations and habitats. Notwithstanding, we consider the publicly available models will enhance the use of camera trap data more broadly and follow their development with great interest.

One of the major advantages of constructing study‐specific DL ensembles is the ability to change the composition to optimise detection performance based on the unique characteristics of the target fauna and study environment. The optimal mix will generally be context specific as the CNNs ingest different semantic (background) information from different settings. For example, in Anschau et al. (2022) where the same DL ensemble approach was used to detect koalas from drone‐acquired thermal imagery, the best‐performing ensembles incorporated YOLO models and Faster‐RCNN models rather than the YOLO‐only ensemble selected for this study. We recognise that the YOLOv5 and Detectron2 models we have used and evaluated in our study are no longer ‘state‐of‐the‐art’, but they are still robust and deliver solid results, and while YOLOv5 was released in 2020 we note that AWC135 utilises EfficientNet‐B5 as its backbone, which is of a similar age having been released in 2019. Of greater importance for those who might wish to test our method, the approach is directly transferable to more recent frameworks and entirely model agnostic. An emerging breed of ‘zero‐shot’ models which are being developed to enable learning to be transferred to unlabelled object classes (Shen et al. 2025) may be a worthy direction of wildlife classification research. Early examples explore wildlife classification via image retrieval, which matches objects with samples in an image database, and via vision‐language models, which match text descriptions generated by large language models with an external knowledge base (Vyskočil and Picek 2025).

Fine‐tuning pre‐trained CNNs reduces the annotation burden significantly. For example, the dataset we processed with our DL ensemble comprised 216,001 images collected over a 5‐month period from 33 sites at Lavinia, Tasmania. To achieve this, we annotated only 3094 fauna images to which we added 760 non‐fauna images with empty annotation files to create a corpus of 3854 annotated images. These images were sourced from a number of the Lavinia sites, as well as from other Tasmanian locations that were part of the deer management program. In reality, the quantum of the Lavinia dataset seen by the ensemble prior to processing is very small (less than 1%). While this does constitute a very small degree of data leakage, in the context of this work this is arguably unavoidable. Quarantining one or two cameras for model training would produce a training corpus that is not representative of the data as a whole, which would compromise model performance. Similarly, excluding cameras that have provided data for model training from further analysis would reduce the coverage of the camera grid, which would compromise the abundance estimation process. The aim of our ensemble approach is to test a method for deriving abundance estimates from DL‐derived outputs for practical applications. Rather than proposing an ‘off‐the‐shelf’ approach that can be deployed anywhere, we are acknowledging that for a lot of scenarios there may be no ‘off‐the‐shelf’ model that is suitable, so we must consider how to get actionable insights with minimal data loss. We note that our approach achieved sound results from a camera grid that was not designed specifically for our study.

Selecting fine‐tuning images from within the dataset to be processed also provides an expedient tool for analysing ‘by‐catch’ data, being fauna other than the original target of the survey (Bruce et al. 2025), such as the macropods we have studied from data collected in a deer monitoring survey. This has particular potential for infrequently monitored fauna that are captured incidentally, such as echidnas, wombats and Tasmanian devils in our dataset. The interrogation of by‐catch data could allow existing CT datasets to be more fully exploited and potentially increase the return on monitoring investment.

We acknowledge that ensemble learning can be computationally expensive and there is a point in terms of ensemble size and complexity where performance gains diminish (Kondratyuk et al. 2020). However, a comparison of processing and memory requirements in this study showed that our ensemble (albeit comprising two YOLOv5 models, one medium and one large) required only 18% more processing time (an additional 1 h and 50 min) and no additional memory than a single medium YOLOv5, whereas a single F‐RCNN with 101 layers was more computationally expensive than both, exceeding ensemble processing time by a further 18% and requiring 18% more memory. We note also that ensemble scale can be adjusted to the compute budget. In the case of the ensemble used in this study, the overhead resulting from the process to load individual images (known as File Input/Output (File I/O)) was only incrementally higher compared to a single model because the data is only loaded once.

## 5 Conclusion

The capacity for ecologists to acquire high quality monitoring data continues to expand as remote sensing technologies advance. The opportunity to elicit deep ecological insights has perhaps never been greater. However retrieving these insights remains difficult, limited in part by an inability to fully exploit the large volumes of data being gathered (Tuia et al. 2022). It will be increasingly important, going forward, for ecologists to pay close attention to developments in computer science, to continually enhance computational, modelling and workflow capabilities (Carey et al. 2019). To this end, we offer our study as an incremental contribution to the pursuit of a fully automated, end‐to‐end wildlife detection and population estimation process.

## Author Contributions

**Grant Hamilton:** conceptualization (supporting), funding acquisition (lead), resources (supporting), supervision (lead), writing – review and editing (supporting). **Greg Hocking:** validation (supporting), writing – review and editing (supporting). **Megan Anschau:** conceptualization (lead), data curation (lead), formal analysis (lead), investigation (lead), methodology (lead), software (equal), validation (lead), visualization (lead), writing – original draft (lead), writing – review and editing (lead). **Simon Denman:** data curation (supporting), resources (supporting), software (equal), supervision (supporting), writing – review and editing (supporting).

## Funding

This work was supported by the Bush Heritage Australia.

## Conflicts of Interest

The authors declare no conflicts of interest.

## Supporting information


**Figure S1:** Precision‐recall curves showing average precision (AP) scores for each fauna class and overall mean‐AP (mAP) for the best‐performing (a) medium and (b) large YOLOv5 v6.0, with performance further illustrated by the confusion matrix for the (c) medium and (d) large YOLOv5 v6.0.
**Table S1:** The automated detection output includes a .txt file containing all predicted wildlife detections.
**Figure S2:** Maps of spatial proxy data representing abundance covariates at camera sites (elevation, slope, vegetation type and distance to nearest water).
**Table S2:** Full list of covariates and data sources used to model the abundance and detection processes affecting Bennett's wallaby and Tasmanian pademelon at Lavinia, King Island, Tasmania, published by DNRET.
**Table S3:** Results of the final model comparison (green line = ΔAIC < 2, red line = cumulative AIC weight < 0.95): there was little apparent difference between the top three models according to the coefficient of determination (*R* ^2^ = 0.86) and AIC (Δ < 2). The top two models were also two of the most parsimonious (9 parameters) of the models that included both state (abundance) and detection processes. Models with no state process performed relatively poorly.
**Table S4:** Performance metrics achieved by the automated classification deep‐learning ensemble based on manual review of the output (NA precision and recall indicate no macropod detections at that site).
**Table S5:** Summary of survey effort and relative abundance indices by site.
**Figure S3:** The plot of predicted abundance by vegetation type shows 52% of detections occurred in scrub‐heath/other natural vegetation (22 sites) compared to 48% in modified land (11 sites). A Pearson's chi‐squared test showed this difference in proportions was significant (chi‐squared = 19.121, df = 1, *p* = 1.23e‐05).
**Figure S4:** Goodness of fit comparison of bootstrap test statistics (tB) from 1000 simulations to original test statistics (t0) for SSE, Pearson's chi‐squared and Freeman Tukey tests. All comparisons returned *p*‐values above 0.05 (SSE: Pr(tB > t0) = 0.302; Pearson's chi‐squared: Pr(tB > t0) = 0.816; Freeman Tukey: Pr(tB > t0) = 0.338).
**Figure S5:** Results of a comparison between automatically generated detection histories subset with classification confidence level thresholds of 0.0 (DH@0.0), 0.5 (DH@0.5), 0.75 (DH@0.75) and 0.8 (DH@0.8) and the detection history derived from full manual review (DH‐MR). ‘T’ (green) indicates a match with a [0] or [1] in DH‐MR while ‘F’ (red) indicates a mismatch and ‘NA’ indicates no data was collected on that occasion.
**Table S6:** Summary of chi‐squared tests of independence comparing number of [0] and [1] label matches per weekly sampling unit between each automatically generated detection history and DH‐MR. Degrees of freedom (df) are calculated from the number of unique values in each vector (df = (*n*‐values(v1)‐1) × (*n*‐values(v2)‐1)).

## Data Availability

Figures S1–S5 and Tables S1–S6 the results of this study are provided in the Supporting Information S1. The scripts used in the workflow are stored in a GitHub repository that can be accessed via Zenodo here (https://zenodo.org/records/21833725).
