# Supplementary material for: Confidence Thresholds: Towards an Automated Workflow for Unmarked Wildlife Population Modelling With Camera Trap Data
Source: Ecol Evol. 2026 Aug 28;16(9):e74227. doi: 10.1002/ece3.74227 (PMC13523075; doi:10.1002/ece3.74227)
Supplement: Supplementary file 1 [file ECE3-16-e74227-s001.docx]

**Confidence thresholds: Towards an automated workflow for unmarked wildlife population modelling with camera trap data**

**Supporting information**

**Fig. S1** Precision-recall curves showing average precision (AP) scores for each fauna class and overall mean-AP (mAP) for the best-performing (a) medium and (b) large YOLOv5 v6.0, with performance further illustrated by the confusion matrix for the (c) medium and (d) large YOLOv5 v6.0.

|  |  |
| --- | --- |
|  |  |

**Table S1** The automated detection output includes a .txt file containing all predicted wildlife detections. We saved the .txt file in .csv format (~145,000 lines) which produced 10 unlabelled columns (a-j), then resaved in .xlxs format to create a recording sheet for manual review, in accordance with Table S1. Consistent treatment of this output ensures a repeatable approach to the manual review so that results flow directly into the scripted workflow.

| **Column** | **Content** | **Action** |
| --- | --- | --- |
| a | Full file path and file name of raw input image | Delete |
| b | Image directory name (eg, “Lavinia_Site_86”) | Rename “site” and retain only the numeric (eg, “86”) |
| c | Full name of input image (eg, “SYCW5333.JPG”) | Rename “frame” and retain only the numeric (eg, “5333”) |
| d | Time stamp providing the date and time (dd/mm/yyyy hh:dd format) of the image capture | Rename “timestamp” |
| e | Class label assigned to the predicted detection signifying the fauna type | Rename “label” |
| f | Certainty value between 0 and 1 | Rename “certainty” |
| g | Pixel coordinates signifying the corners of the bounding box surrounding the predicted detection | Rename “bb1” |
| h | Pixel coordinates signifying the corners of the bounding box surrounding the predicted detection | Rename “bb2” |
| i | Pixel coordinates signifying the corners of the bounding box surrounding the predicted detection | Rename “bb3” |
| j | Pixel coordinates signifying the corners of the bounding box surrounding the predicted detection | Rename “bb4” |
| Insert 2 new columns between e-f | Blank column for recording the manual review decision (true positive, false positive, false negative, duplicate, misclassification) | Name “description” |
|  | Blank column for recording any pertinent observations (not always used) | Name “note” |

**Fig. S2** Maps of spatial proxy data representing abundance covariates at camera sites (elevation, slope, vegetation type and distance to nearest water).

|  |  |
| --- | --- |
|  |  |

**Table S2** Full list of covariates and data sources used to model the abundance and detection processes affecting Bennett’s wallaby and Tasmanian pademelon at Lavinia, King Island, Tasmania, published by DNRET.

| **Covariate** | **Process** | **Source** |
| --- | --- | --- |
| Distance to water | Abundance | LIST Hydrographic Lines – King Island Municipality |
| Elevation | Abundance | LIST Tasmania 25 Metre Digital Elevation Model – King Island Municipality |
| Slope | Abundance | LIST Contours 10m – King Island Municipality |
| Vegetation type | Abundance + detection | NCH Tasveg 4.0 |
| Minimum temperature in sampling week | Detection | Australian Government Bureau of Meteorology daily minimum temperature – King Island Airport |
| Maximum temperature in sampling week | Detection | Australian Government Bureau of Meteorology daily maximum temperature – King Island Airport |
| Rainfall total in sampling week | Detection | Australian Government Bureau of Meteorology daily rainfall – Naracoopa |
| Survey effort | Detection | Operational CT days in sampling week |
| Week number | Detection | Week number based on International Organisation for Standardisation rule ISO-8601 |

**Table S3** Results of the final model comparison (green line = ΔAIC <2, red line = cumulative AIC weight <0.95): there was little apparent difference between the top three models according to the coefficient of determination (R^2^ = 0.86) and AIC (Δ<2). The top two models were also two of the most parsimonious (9 parameters) of the models that included both state (abundance) and detection processes. Models with no state process performed relatively poorly.

| **Model** | **nParameters** | **AIC** | **ΔAIC** | **AIC weight** | **R-squared** | **Cumul. AIC weight** |
| --- | --- | --- | --- | --- | --- | --- |
| N ~ water + elevation + slope + veg  p ~ min temp + effort + veg | 9 | 148.99 | 0.00 | 0.27 | 0.86 | 0.27 |
| N ~ water + elevation + slope + veg  p ~ rain + effort + veg | 9 | 149.40 | 0.41 | 0.22 | 0.86 | 0.48 |
| N ~ water + elevation + slope + veg  p ~ min temp + rain + effort + veg | 10 | 150.75 | 1.75 | 0.11 | 0.86 | 0.59 |
| N ~ water + elevation * slope + veg  p ~ min temp + rain + effort | 10 | 151.42 | 2.43 | 0.08 | 0.86 | 0.67 |
| N ~ water + elevation + slope + veg  p ~ min temp * rain + effort | 10 | 151.45 | 2.46 | 0.08 | 0.86 | 0.75 |
| N ~ water + elevation + slope + veg  p ~ min temp + rain + effort | 9 | 151.52 | 2.52 | 0.08 | 0.85 | 0.82 |
| N ~ water + elevation + slope + veg  p ~ min temp + rain + effort + week + veg | 11 | 152.62 | 3.63 | 0.04 | 0.86 | 0.87 |
| N ~ water + elevation * slope + veg  p ~ min temp + rain + effort + week | 11 | 153.19 | 4.19 | 0.03 | 0.86 | 0.90 |
| N ~ water + elevation + slope + veg  p ~ min temp + rain + effort + week | 10 | 153.21 | 4.21 | 0.03 | 0.85 | 0.93 |
| N ~ water + elevation + slope + veg  p ~ min temp * rain + effort + week | 11 | 153.45 | 4.46 | 0.03 | 0.86 | 0.96 |
| N ~ 1  p ~ min temp + max temp + rain + effort + week | 7 | 153.73 | 4.73 | 0.02 | 0.82 | 0.99 |
| N ~ water + elevation + slope + veg  p ~ min temp + max temp + rain + effort + week | 11 | 154.83 | 5.84 | 0.01 | 0.85 | 1.00 |
| N ~ 1  p ~ 1 | 2 | 199.67 | 50.68 | 0.00 | 0.00 | 1.00 |
| N ~ water + elevation + slope + veg  p ~ 1 | 6 | 204.86 | 55.87 | 0.00 | 0.08 | 1.00 |

**Table S4** Performance metrics achieved by the automated classification deep-learning ensemble based on manual review of the output (NA precision and recall indicate no macropod detections at that site).

| **Site** | **Accuracy** | **Precision** | **Recall** | **True**  **positive** | **False**  **positive** | **Duplicate** | **Misclass-**  **ification** | **False**  **negative** |
| --- | --- | --- | --- | --- | --- | --- | --- | --- |
| **4** | 1.00 | 1.00 | 0.91 | 20 | 0 | 2 | 1 | 1 |
| **7** | 0.75 | 0.99 | 0.56 | 1156 | 14 | 33 | 148 | 747 |
| **15** | 0.99 | 0.75 | 0.72 | 123 | 42 | 0 | 20 | 27 |
| **16** | 0.96 | 0.89 | 0.53 | 164 | 21 | 0 | 37 | 107 |
| **22** | 1.00 | 0.92 | 0.75 | 12 | 1 | 0 | 1 | 3 |
| **24** | 1.00 | 1.00 | 0.75 | 3 | 0 | 0 | 1 | 0 |
| **25** | 0.66 | 0.97 | 0.45 | 1030 | 35 | 1 | 317 | 945 |
| **59** | 0.98 | 0.99 | 0.44 | 139 | 2 | 6 | 0 | 177 |
| **63** | 0.80 | 0.91 | 0.15 | 109 | 11 | 3 | 0 | 632 |
| **65** | 0.67 | 1.00 | 0.55 | 86 | 0 | 3 | 0 | 71 |
| **66** | 0.83 | 0.94 | 0.61 | 201 | 12 | 9 | 0 | 131 |
| **67** | 0.95 | 0.25 | 1.00 | 1 | 3 | 0 | 0 | 0 |
| **68** | 0.98 | 0.15 | 0.45 | 30 | 174 | 4 | 0 | 37 |
| **69** | 0.91 | 0.79 | 0.73 | 19 | 5 | 1 | 0 | 7 |
| **72** | 0.65 | 1.00 | 0.62 | 1120 | 2 | 28 | 0 | 699 |
| **74** | 0.97 | 0.67 | 0.55 | 29 | 14 | 0 | 0 | 24 |
| **75** | 0.68 | 0.91 | 0.49 | 43 | 4 | 0 | 1 | 44 |
| **77** | 0.87 | 0.88 | 0.68 | 52 | 7 | 0 | 0 | 25 |
| **78** | 0.76 | 0.87 | 0.39 | 266 | 40 | 13 | 0 | 416 |
| **81** | 0.99 | NA | NA | 0 | 61 | 0 | 0 | 0 |
| **84** | 0.99 | NA | NA | 0 | 2 | 0 | 0 | 0 |
| **85** | 0.82 | 0.70 | 0.23 | 129 | 56 | 5 | 0 | 431 |
| **88** | 0.74 | 0.75 | 0.20 | 15 | 5 | 1 | 2 | 58 |
| **90** | 0.95 | 0.73 | 0.46 | 311 | 114 | 16 | 11 | 352 |
| **91** | 0.95 | 0.91 | 0.45 | 108 | 11 | 6 | 0 | 131 |
| **94** | 0.97 | NA | NA | 0 | 41 | 0 | 0 | 0 |
| **95** | 0.98 | 0.98 | 0.63 | 125 | 3 | 6 | 0 | 73 |
| **96** | 0.98 | 0.30 | 0.20 | 21 | 49 | 0 | 0 | 86 |
| **106** | 0.98 | NA | NA | 0 | 23 | 0 | 0 | 0 |
| **119** | 0.99 | 1.00 | 0.41 | 100 | 0 | 3 | 0 | 143 |
| **120** | 0.99 | 0.90 | 0.49 | 69 | 8 | 3 | 0 | 71 |
| **122** | 0.97 | 0.77 | 0.55 | 33 | 10 | 2 | 0 | 27 |
| **126** | 0.96 | 0.96 | 0.79 | 26 | 1 | 0 | 0 | 7 |

**Table S5** Summary of survey effort and relative abundance indices by site.

| **site** | **Start date** | **End date** | ***Early end reason** | **Independent**  **Observations** | **Operational**  **camera days** | **RAI** |
| --- | --- | --- | --- | --- | --- | --- |
| 4 | 20/10/2022 | 4/12/2022 |  | 12 | 46 | 0.3 |
| 7 | 18/10/2022 | 4/12/2022 |  | 389 | 48 | 8.1 |
| 15 | 18/10/2022 | 28/11/2022* | Full card (vegetation) | 82 | 42 | 2.0 |
| 16 | 18/10/2022 | 4/12/2022 |  | 66 | 48 | 1.4 |
| 22 | 20/10/2022 | 16/11/2022* | Flat + full card (vegetation) | 7 | 28 | 0.3 |
| 24 | 18/10/2022 | 4/12/2022 |  | 4 | 48 | 0.1 |
| 25 | 19/10/2022 | 4/12/2022 |  | 289 | 47 | 6.1 |
| 59 | 19/10/2022 | 22/11/2022* | Full card (vegetation) | 81 | 35 | 2.3 |
| 63 | 17/10/2022 | 14/11/2022* | Flat battery | 105 | 29 | 3.6 |
| 65 | 17/10/2022 | 28/10/2022* | Flat battery | 44 | 12 | 3.7 |
| 66 | 18/10/2022 | 4/12/2022 |  | 141 | 48 | 2.9 |
| 67 | 18/10/2022 | 4/12/2022 |  | 1 | 48 | 0.0 |
| 68 | 18/10/2022 | 19/10/2022* | Full card (shadows) | 3 | 2 | 1.5 |
| 69 | 17/10/2022 | 4/12/2022 |  | 17 | 49 | 0.3 |
| 72 | 20/10/2022 | 4/12/2022 |  | 288 | 46 | 6.3 |
| 74 | 20/10/2022 | 4/12/2022 |  | 40 | 46 | 0.9 |
| 75 | 19/10/2022 | 12/11/2022* | Not flat / card not full | 43 | 25 | 1.7 |
| 77 | 19/10/2022 | 4/12/2022 |  | 46 | 47 | 1.0 |
| 78 | 18/10/2022 | 4/12/2022 |  | 208 | 48 | 4.3 |
| 81 | 17/10/2022 | 27/10/2022* | Flat + full card (cows) | 0 | 11 | 0.0 |
| 84 | 20/10/2022 | 27/10/2022* | Not flat / card not full | 0 | 8 | 0.0 |
| 85 | 17/10/2022 | 4/12/2022 |  | 152 | 49 | 3.1 |
| 88 | 18/10/2022 | 4/12/2022 |  | 40 | 48 | 0.8 |
| 90 | 17/10/2022 | 4/12/2022 |  | 153 | 49 | 3.1 |
| 91 | 17/10/2022 | 4/12/2022 |  | 76 | 49 | 1.6 |
| 94 | 17/10/2022 | 4/12/2022 |  | 0 | 49 | 0.0 |
| 95 | 20/10/2022 | 4/12/2022 |  | 41 | 46 | 0.9 |
| 96 | 17/10/2022 | 4/12/2022 |  | 26 | 49 | 0.5 |
| 106 | 17/10/2022 | 23/10/2022* | Not flat / card not full | 0 | 7 | 0.0 |
| 119 | 19/10/2022 | 19/11/2022* | Full card (vegetation) | 34 | 32 | 1.1 |
| 120 | 17/10/2022 | 4/12/2022 |  | 40 | 49 | 0.8 |
| 122 | 17/10/2022 | 4/12/2022 |  | 25 | 49 | 0.5 |
| 126 | 17/10/2022 | 4/12/2022 |  | 24 | 49 | 0.5 |

**Fig. S3** The plot of predicted abundance by vegetation type shows 52% of detections occurred in scrub-heath / other natural vegetation (22 sites) compared to 48% in modified land (11 sites). A Pearson’s chi-squared test showed this difference in proportions was significant (chi-squared=19.121, df=1, p=1.23e-05).

**Fig. S4** Goodness of fit comparison of bootstrap test statistics (tB) from 1000 simulations to original test statistics (t0) for SSE, Pearson's chi-squared and Freeman Tukey tests. All comparisons returned p-values above 0.05 (SSE: Pr(tB > t0)=0.302; Pearson's chi-squared: Pr(tB > t0)=0.816; Freeman Tukey: Pr(tB > t0)=0.338).

**Fig. S5** Results of a comparison between automatically generated detection histories subset with classification confidence level thresholds of 0.0 (DH@0.0), 0.5 (DH@0.5), 0.75 (DH@0.75) and 0.8 (DH@0.8) and the detection history derived from full manual review (DH-MR). “T” (green) indicates a match with a [0] or [1] in DH-MR while “F” (red) indicates a mismatch and “NA” indicates no data was collected on that occasion.

| **DH@0.0** | **DH@0.5** | **DH@0.75** | **DH@0.8** |
| --- | --- | --- | --- |
|  |  |  |  |

**Table S6** Summary of chi-squared tests of independence comparing number of [0] and [1] label matches per weekly sampling unit between each automatically generated detection history and DH-MR. Degrees of freedom (df) are calculated from the number of unique values in each vector (df = (n-values(v1)-1) * (n-values(v2)-1)).

| **Detection histories compared** | **χ-squared** | **df** | **p-value** |
| --- | --- | --- | --- |
| DH@0.0 : DH@0.5 | 35 | 30 | 0.2426 |
| DH@0.0 : DH@0.75 | 22.75 | 20 | 0.3012 |
| DH@0.0 : DH@0.8 | 28 | 25 | 0.3079 |
| DH@0.5 : DH@0.75 | 28 | 24 | 0.2600 |
| DH@0.5 : DH@0.8 | 35 | 30 | 0.2426 |
| DH@0.75 : DH@0.8 | 28 | 20 | 0.1094 |
